# DORA: an interactive map for the visualization and analysis of ancient human DNA and associated data

**DOI:** 10.1093/nar/gkae373

**Published:** 2024-05-14

**Authors:** Keith D Harris, Gili Greenbaum

**Affiliations:** Department of Ecology, Evolution and Behavior, The Hebrew University of Jerusalem, Givat Ram, 9190401 Jerusalem, Israel; Department of Ecology, Evolution and Behavior, The Hebrew University of Jerusalem, Givat Ram, 9190401 Jerusalem, Israel

## Abstract

The ability to sequence ancient genomes has revolutionized the way we study evolutionary history by providing access to the most important aspect of evolution—time. Until recently, studying human demography, ecology, biology, and history using population genomic inference relied on contemporary genomic datasets. Over the past decade, the availability of human ancient DNA (aDNA) has increased rapidly, almost doubling every year, opening the way for spatiotemporal studies of ancient human populations. However, the multidimensionality of aDNA, with genotypes having temporal, spatial and genomic coordinates, and integrating multiple sources of data, poses a challenge for developing meta-analyses pipelines. To address this challenge, we developed a publicly-available interactive tool, DORA, which integrates multiple data types, genomic and non-genomic, in a unified interface. This web-based tool enables browsing sample metadata alongside additional layers of information, such as population structure, climatic data, and unpublished samples. Users can perform analyses on genotypes of these samples, or export sample subsets for external analyses. DORA integrates analyses and visualizations in a single intuitive interface, resolving the technical issues of combining datasets from different sources and formats, and allowing researchers to focus on the scientific questions that can be addressed through analysis of aDNA datasets.

## Graphical Abstract

**Graphical Abstract:**
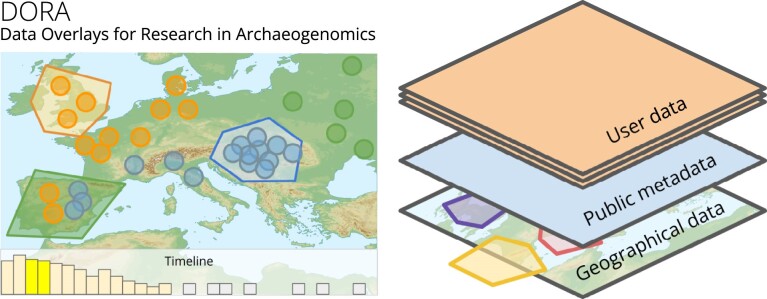

## Introduction

Until recently, answering questions regarding evolution and demographics of human history has relied on inference from contemporary sequenced genomes and a small number of sequenced ancient genomes (1). Over the past decade, the availability of human ancient DNA (aDNA) has increased rapidly, almost doubling every year (2). These data include high-quality whole genome shotgun sequencing for a very small number of samples of interest, low coverage whole genome shotgun sequencing, and sequence capture targeting SNPs in specific SNP panels (e.g. Human Origins, 1240K (3,4)). These resources now allow for testing evolutionary questions directly, rather than relying on indirect inference, by tracking changes in allele frequencies, genomic compositions, and phenotypic predictions over many millennia (5–8).

The visualization and analysis of aDNA is challenging, partly owing to the multi-dimensionality of the individual data points. For every genotype from an individual sample in a dataset, there is a (i) genomic position, (ii) geographic location, and (iii) temporal position (sample dating, which also varies between dating methods). Different analyses can consider a subset of genotypes along windows in each of these dimensions. Selection of such windows can be based on geographic regions of interest, time periods of interest, and also on accessory data such as sample quality, specified regions of the genome, climatic databases, geographical boundaries, and other considerations relevant to the specific research question. One of the challenges of meta-analyses of ancient human genomes is the integration and visualization of these multiple sources of data, which is necessary to allow selection of the specific genotypes to be analyzed. Such selection procedures are often informed by exploratory analyses of the dataset. Tools have been developed that are specifically designed to visualize the results of analyses of aDNA (9), but these require additional tools in order to generate results. Ideally, the first steps of such meta-analyses would be conducted within a single framework that would allow exploration of the dataset by allowing intuitive selection of spatial, temporal, and genomic windows of genotypes, along with population genetic analyses of the selected genotypes.

Here, we present an interactive map of human aDNA samples, called **D**ata **O**verlays for **R**esearch in **A**rchaeogenomics (DORA). This tool visualizes aDNA samples on a geographic map, and allows selection of samples by visualizing the characteristics of the samples (e.g. metadata of the sample, such as coverage). It also includes additional data layers that can be displayed alongside the samples, such as climatic data and results of downstream population structure analyses. Importantly, the tool is designed to allow users to integrate these data layers in order to select subsets of samples according to geographic regions that can be drawn on the map, and temporal windows that can be selected using the slider under the map. These subsets can be exported, or used in meta-analyses through the DORA interface. The interface is web-based and its resources are stored on Amazon Web Services Simple Storage Service (AWS S3), while private user data added to the tool will be available only on the local machine and not uploaded to AWS S3. DORA can facilitate a range of meta-analyses of ancient genomes and will allow users to seamlessly integrate published resources with their unpublished genomic samples or other data layers.

To demonstrate the usefulness of this tool, we have pre-loaded a curated aDNA dataset, the *Allen Ancient DNA Resource* (AADR) (2), along with the TraCE21K climatic data from the database *Climatologies at high resolution for the earth’s land surface areas* (CHELSA) (10), and ADMIXTURE results for ancient samples in the AADR. We also developed an interface for loading polygenic scores (PGSs), which can be used to predict the phenotypes of the ancient samples based on their genomes, from the PGSCatalog database (11); DORA is designed to directly apply PGSs to the aDNA samples loaded into the tool. We demonstrate some of the analyses that can be performed with the tool, including tracking allele frequencies in geographically defined populations, computing temporal trends in genetic differentiation between populations using pairwise *F*_*ST*_ and PCA, and tracking polygenic score values of individuals and demarcated populations over time. DORA integrates this variety of data types, analyses and visualizations, in a single intuitive interface, resolving the technical issues of combining datasets from different sources and formats, and allowing researchers to focus on the analysis and the scientific questions of interest.

## Features of DORA

**Figure 1.**
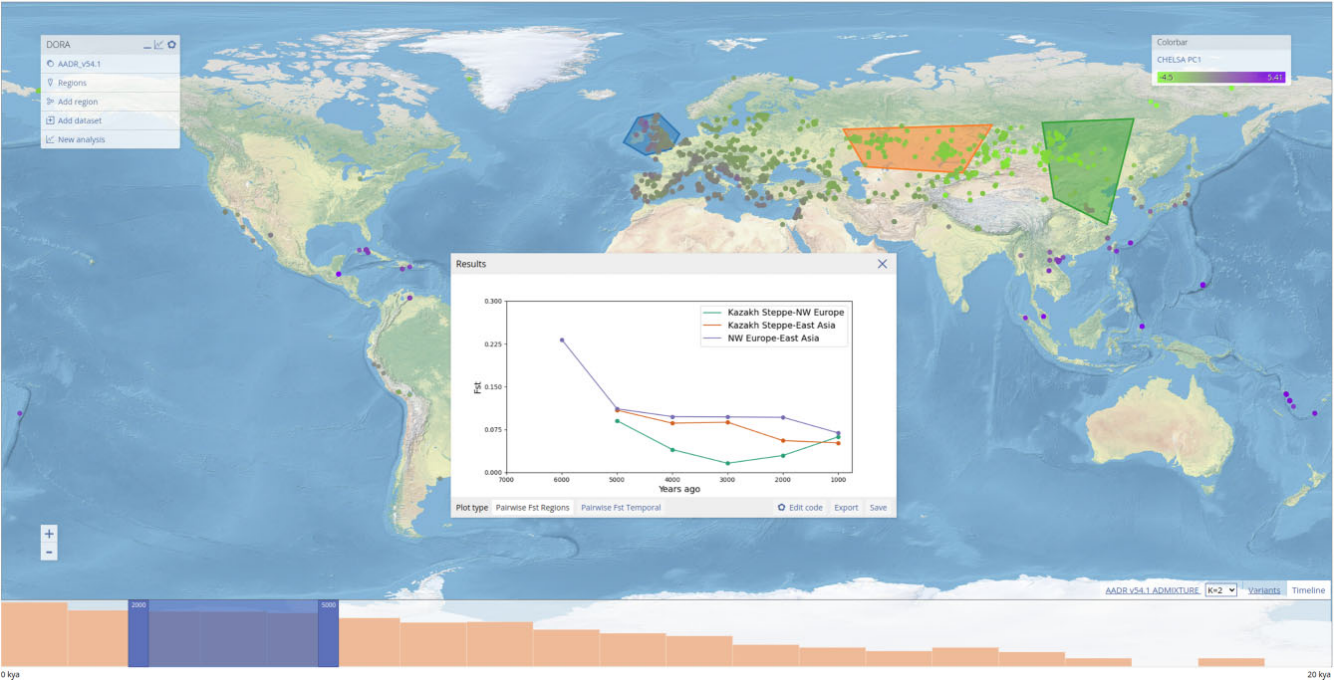
Main interface of DORA. Layers are shown in the main menu on the top left corner. Samples appear as dots on the map, with the color reflecting a specific attribute of the sample, which can be selected on the ‘Colorbar’ panel on the right. In this example, we selected the ‘CHELSA PC1’ attribute, which reflects predicted climate differences between samples based on their geographic location and the archaeological dating of the sample (i.e. the climate at the time the individual lived). At the bottom of the map is a timeline of sample data within the displayed geographic boundaries, and the variants panel, that can be opened by clicking the ‘Variants’ tab. The blue highlighted bins indicate the year range of displayed samples and can be moved to select specific ranges. In this example, the results of a pairwise *F*_*ST*_ analysis of selected geographic regions are plotted (for details, see the *Example analyses* section).

The main display of DORA is a world map on top of which data is visualized. By default, DORA loads a topographical map of the world in equirectangular projection (12) and sample metadata (a screenshot of the main display with metadata loaded from AADR is shown in Figure 1). Samples are represented as dots on the map, with the color of the dots reflecting additional sample metadata, such as coverage or dating, as defined in the ‘Colormap’ box on the right. The map can be browsed spatially by zooming in or out, or dragging the map with the cursor. At the bottom of the map is a timeline that shows the number of available samples in predefined temporal bins (in 100s of years). A slider on the timeline with a blue transparent background defines which samples will be displayed on the map; users can shift the sides of the slider to define this window. In addition, a ‘variants panel’ can be opened by clicking on the ‘Variants’ tab at the bottom right of the screen. This displays selected variants according to their genomic regions; new genomic regions can be added by using the variants form that appears when clicking on the variant tab, while selected genomic regions can be removed by highlighting them in the variants form and clicking ‘Delete’. Variant selection in this panel is used for analyses that are run on multiple loci, such as *F*_*ST*_ or PCA (see the *Example analyses* section below).

To select samples according to their geographic positions, users can define ‘Regions’, which are colored polygons that demarcate samples according to their geographic coordinates for subsequent analyses or export. Geographic regions are created by clicking ‘Add region’ on the main menu. Once clicked, the next successive left-clicks on the map will create vertices of the polygon. Right-clicking will save the polygon with the marked vertices. Geographic regions are stored in the browser cache until deleted; these regions can be deleted by clicking a region and pressing the delete key. Clicking on a geographic region will also allow the user to edit the region label, export sample metadata, or exclude samples in the region from analyses.

### Adding datasets

**Figure 2.**
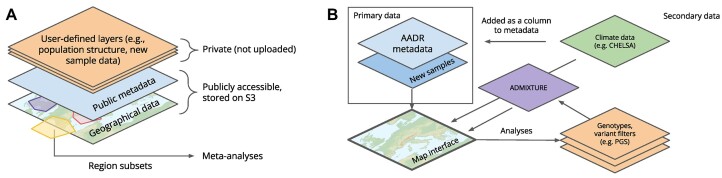
Schematic representation of the organization of data layers in DORA. (**A**) Pre-loaded data includes publicly available datasets, including the AADR and CHELSA TraCE21K. Additional user-defined layers can be added by users, and will be accessible only on the local machine of the user. Analyses can be conducted on all available data. (**B**) Primary data includes files that provide mandatory metadata of samples, including a date estimate and geolocation. Accessory metadata can be added from a variety of sources, such as climate data for sample locations or ADMIXTURE results. These data can then be displayed on the map, used to filter individuals for analyses, or plotted along with the results of genetic analyses.

DORA overlays multiple data sources onto a single map display, allowing users to combine these sources when selecting samples for subsequent analyses (Figure 2). Layers that were pre-loaded to demonstrate the features of DORA will load by default, including the map (based on *Natural Earth* (12)), aDNA metadata and genotypes from the AADR (2), CHELSA climate model data for the past 20 000 years (10), and ADMIXTURE results for ancient samples in the AADR. Additional datasets can be added by clicking the ‘Add dataset’ button; these can either add samples to the map (primary datasets; see scheme in Figure 2B), or add metadata for existing samples, according to their sample IDs (Table 1). If samples have multiple possible attributes to display, the user is able to select the desired attribute to color the samples using the ‘Colormap’ menu near the top right corner of the map.

**Table 1.** Data types supported by DORA

Data type	Use case	Formats
Samples	Displaying additional samples on the map with associated metadata	CSV/TSV
Metadata	Displaying additional metadata for samples on the map, such as climate information or archaeological data	CSV/TSV
Genotypes	Analysis of unpublished genetic data, adding imputed genotypes	PLINK BED
Structure results	Displaying population structure of AADR or additional samples on the map	PLINK FAM+Q
PGS	Predicting traits of loaded samples	PGSCatalog API

### Analyses

DORA allows users to conduct meta-analyses on the genetic data loaded into the interface, which includes the AADR genotypes loaded into DORA by default, and any other data loaded into the browser (in PLINK BED format). One of the main benefits of running analyses from the map interface is that users can integrate multiple data sources when defining subsets of samples, and run a variety of analyses on these subsets from the same interface. Analyses can be initiated by clicking the ‘New analysis’ button. Analyses are run on the selected geographic regions, in temporal windows assigned according to the timeline or in the analysis dialog box field ‘Temporal range (in years)’, and on genomic regions as defined in the variants panel (except the allele frequency analysis, which runs on a single variant specified in the analysis dialog box). Additional filtering criteria for samples and variants can be specified in the analysis dialog box. The available analyses include: (i) allele frequency analysis, which shows the temporal trajectory of the allele frequency of a specific variant; (ii) expected heterozygosity of selected genetic variants; (iii) pairwise *F*_*ST*_ between all the pairs of geographic regions (using the implementation of Hudson *F*_*ST*_ estimation (13) from scikit-allel (14)); (iv) PCA of samples from all geographic regions; and (v) computed polygenic scores of samples in all geographic regions, over the chosen temporal windows (PGSs must first be imported from PGSCatalog).

Analyses are run on AWS Lambda for publicly available data, and on the local machine for locally stored data. DORA uses Pyodide (15) to provide the same Python analysis environment in the browser and on AWS Lambda. The analysis script is loaded by the specific environment, and the BED file is read either from AWS S3 (in the case of AWS Lambda) or from the browser cache (in the case of local data). The user receives a link to the analysis results when the analysis is run; this link will display the results as soon as they are ready. When the analysis is complete, the results are also processed and plotted using Python code run using Pyodide. Each analysis comes with a number of pre-defined plotting formats, such as line plots or scatter plots; this Python code can be manipulated by the user by clicking ‘Edit code’, and the figure updated using the ‘Update’ button (Figure 1). All analysis plots presented here were generated by the analysis feature of DORA.

## Example analyses

**Figure 3.**
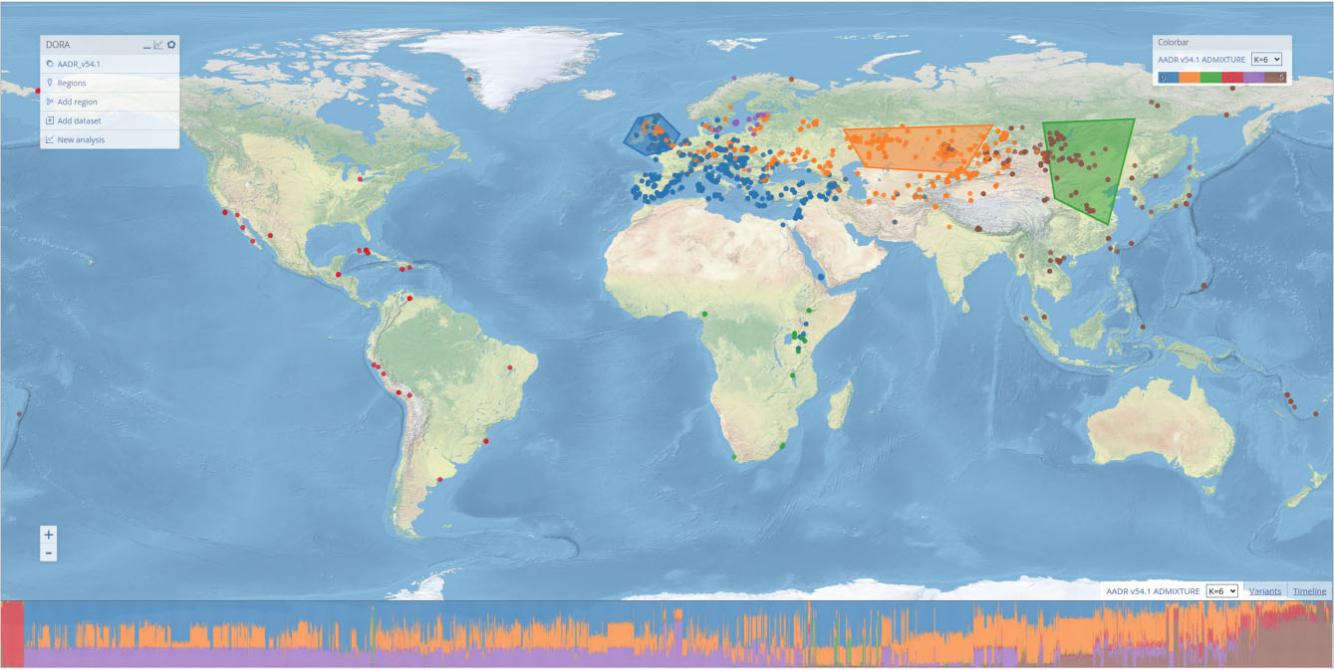
Selection of geographic regions based on population structure analysis. ADMIXTURE results are plotted on the map, with samples colored according to their largest ancestry component. This can be selected using the ‘Colorbar’ menu on the top right, using the pre-loaded ADMIXTURE results, or after adding user-generated ADMIXTURE results (see *Adding datasets*). The STRUCTURE-plot for all samples in the selected window is at the bottom of the screen, ordered according to longitude. Geographic regions have been manually selected on the map to roughly correspond to the plotted ADMIXTURE results.

To illustrate some potential uses of DORA, we present here example analyses that utilize different features of the tool. These tasks did not require the preparation of data with external tools and analyses were completed within 1–5 min. The results are also processed and plotted by DORA, so that the entire task is completed using the same interface. In these examples, we used a previously conducted population structure analysis to select geographic regions, conducted with ADMIXTURE (16). The ADMIXTURE results are represented in Figure 3 as colored samples on the map, where each individual is colored by its main ancestry component. At the bottom of the map, above the timeline, a ‘STRUCTURE-plot’ is shown for all displayed samples. Based on these results, we drew three polygons representing Northwest Europe, the Kazakh Steppe and East Asia.

### Allele frequency fluctuations over time in different populations

**Figure 4.**
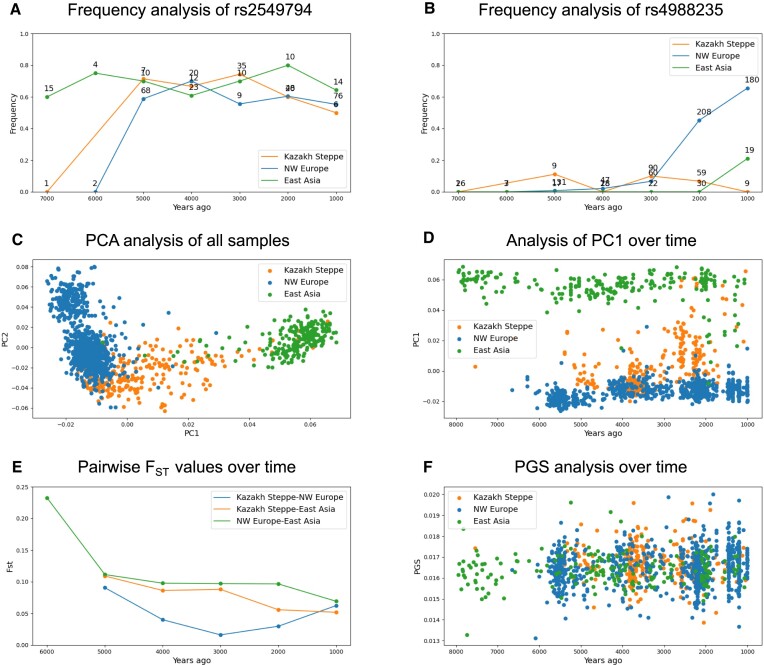
Example analyses run with DORA. Using the polygon generation tool, we defined three geographic regions (Northwest Europe in blue, the Kazakh Steppe in orange, and East Asia in green). We ran different analyses on these regions. (**A**) Temporal changes in allele frequency of variant rs2549794 (previously suggested to be related to the Black Death). (**B**) Temporal changes in allele frequency of variant rs4988235 (previously suggested to be related to lactase persistence). (**C**) PCA of samples from all temporal windows in all three geographic regions for a subset of variants from chromosome 1. (**D**) PC1 of the three geographic regions over time using the same PCA from panel (C). (**E**) Pairwise *F*_*ST*_ values between the three regions over time, using the same subset of variants from panel (C). (**F**) Height PGS (computed using data integrated from the PGSCatalog) for samples in all three geographic regions over time (PGSCatalog ID: PGS000297).

Tracking allele frequencies over time is a simple analysis that is important in order to identify evolutionary processes. Such analyses can be performed by DORA in the selected geographic and temporal windows. We selected two variants that are at intermediate frequencies in modern populations: a variant that was suggested to have undergone positive selection during the Black Plague (8,17) (rs2549794), and an allele that has been suggested to confer lactase persistence (rs4988235). In the temporal range selected, the frequency of the non-reference allele of the rs2549794 variant shows no clear trend in any geographic region (Figure 4A), whereas rs4988235 increases monotonically in the NW Europe and East Asia geographic regions (Figure 4B), an increase previously attributed to positive selection (18). Such analyses may be useful to examine whether observed trends are common or if they are indeed consistent with selection processes.

### PCA of samples in selected geographic regions

DORA can run PCA on a subset of samples, for specified genomic regions. By default, DORA pools samples from all windows and runs the PCA on all selected variants. To deal with missing data, DORA includes different methods that can be selected in the analysis dialog box. By default DORA uses EMU, which is designed for datasets with high levels of missing data (19); alternatively, missing genotypes can be imputed using the allele frequency of all subsets or of the same subset, or setting the frequency as 0. We show here two possible displays of PCA results generated in DORA using EMU: a scatter plot of the first two principal components (Figure 4C), and a scatter plot of the temporal position of the samples and the first principal component (Figure 4D).

In the standard PCA plot, geographic region selection captures genetic differences between the samples located in these regions (Figure 4C). Overlap can be seen mainly between the Kazakh Steppe and Northwest Europe, but also between East Asia and the Kazakh Steppe. To see whether this overlap occurs in specific temporal windows, we can plot the dating of individual samples against the first principal component: here the separation of samples from different geographic regions is apparent in each selected period, with increasing overlap between East Asia and the Kazakh Steppe closer to the present (Figure 4D).

### Computing pairwise *F*_*ST*_ values for geographic region pairs

We can also visualize genetic differentiation at a population-level using the *F*_*ST*_ statistic. DORA can compute pairwise *F*_*ST*_ values for groups of samples defined by geographic region, temporal window, and subsets of genetic variants. As an analog to (Figure 4D), we computed pairwise *F*_*ST*_ values for pairs of geographic regions within each temporal window (Figure 4E).

The results show high genetic differentiation between the three geographic regions that decreases towards the present, with a drastic decrease in *F*_*ST*_ around the period of the Yamnaya expansion (4000–5000ya) (20). It is important to note that *F*_*ST*_ values are also affected by the aspects of the scenario such as the number of samples used to calculate the *F*_*ST*_ value in each window, population structure, and within-population variance (21), which may also affect the *F*_*ST*_ values. The higher *F*_*ST*_ value for the Kazakh Steppe pairwise values in the 1000–2000ya temporal window can most likely be attributed to different sample sizes in this window. Such pairwise *F*_*ST*_ analyses can be used together with population structure analyses as a means to define distinct geographic regions that show genetic differentiation, before running downstream analyses.

### Applying polygenic scores (PGSs) to aDNA samples

Polygenic scores (PGSs) are used to compute predicted trait values for an individual by weighting variants according to their contribution to the trait. Applying PGSs that have been developed using contemporary GWAS to aDNA samples provides an interesting opportunity to predict phenotypically meaningful genetic changes beyond single variants. In some cases, predicted traits can be correlated with measurable phenotypes, if they can be inferred from skeletal remains (7). To allow crosstalk between the growing number of PGSs in the curated PGSCatalog (11), and aDNA samples, we added a feature to DORA that allows users to import PGSs directly from PGSCatalog, using a simple search interface. Users can search for a PGS according to the trait of interest, and the selected PGS will be processed to be compatible with genotypic data loaded into DORA.

To demonstrate this feature, we present the results of applying a PGS of height, which was developed based on the UK Biobank (22), to aDNA samples in the three geographic regions. As with PCA results, the results of the PGS analysis can be displayed alongside other metadata of the samples, including their dating, coverage, climate, or additional user-defined metadata. We see no clear differences in the prediction of height between geographic regions or temporal windows according to this PGS analysis (Figure 4F). The application of PGSs to aDNA samples is not trivial due to the low transferability of PGSs between genetically distant populations (23). Nevertheless, the exploration of temporal changes in predicted trait values for different traits in different geographic regions could provide an important lens through which human evolution can be studied; DORA not only simplifies conducting such analyses, it also allows integrating it with environmental data, potentially allowing for identification of drivers of such phenotypic changes.

## Discussion

We present here DORA, a web-based tool for exploratory analysis of aDNA data. To demonstrate the features of this tool, we pre-loaded publicly available datasets and conducted a number of example analyses. These analyses were completed using the same interface and without using external tools. More elaborate analyses can be conducted with additional data layers.

The goal of DORA is to make the growing body of published human aDNA datasets more accessible for visualization and analysis, and to make the integration of accessory datasets for selection of samples and correlation with genetic analyses more intuitive. DORA combines serverless cloud-compute for analysis of publicly available datasets with local, browser-based analysis of private data. The results of these analyses are displayed in the browser, and their display can be manipulated by the user. Using the same Python environment for cloud-based and local computation, in addition to the generation of plots, makes the analysis pipelines on DORA flexible, transparent and accessible to users, and supports straightforward development of additional analyses.

Interface-based tools are particularly important for aDNA studies, because the multidimensionality of the data can pose a challenge to defining comprehensive and coherent criteria for selecting samples and genotypes for analyses. Downstream analyses also regularly involve multiple computational tools, each of which can have different requirements for the input format of the data. With currently available tools, such as PLINK and Python map plotting libraries, the visualization and analysis of aDNA involves multiple steps and libraries, and is not interactive; in DORA, these can be combined into a single step, and performed from the same interface.

We envision this tool as an important resource for researchers in the field of human aDNA by making accessible analyses that combine multiple existing resources with population genetics tools. This will allow rapid and intuitive definition of analyses and exploration of published aDNA data alongside unpublished samples.

## Data Availability

DORA can be accessed at https://dora.modelrxiv.org, and its code is available at https://github.com/carrowkeel/dora and https://doi.org/10.6084/m9.figshare.25304920.v2. The latest version of the AADR is accessible at doi.org/10.7910/DVN/FFIDCW. The topographic map can be downloaded from Natural Earth (12). The environmental variables used to compute the CHELSA PCs can be downloaded from the CHELSA website (https://chelsa-climate.org/chelsa-trace21k). PGSs used can be downloaded from PGSCatalog (https://www.pgscatalog.org).
